# God and the Robin

**Published:** 1887-01

**Authors:** 


### GOD AND THE ROBIN.

We extract the following exquisite allegory, under the above title, from a little pamphlet collection of "Papers in Penology," relating to Prison management. It was formerly contributed to *The Summary*, the reformatory newspaper, of the New York State Reformatory at Elmira, by a youthful burglar who had once been one of the foremost skeptics among his prison-mates:

"Early in the morning before the lazy cock crows, you may hear the robin singing his welcome to the sun. All is quiet till his music rends the air, and as you listen you are inspired with thoughts of Him who made the robin and you. Perhaps the sweet song is a prayer of thanks to God for sheltering them from the dangers of the night. Do they know of God? Who can tell? Perhaps He is the cause of what we in our ignorance call instinct. Once, as I listened to their music, I fell asleep, and dreamt of a house near the sea. It had a lawn in front on which was a robin hopping in search of food for her young. But as she hopped about, the sky seemed to grow darker. I knew that a storm was approaching, and when it came, I saw the robin cling to the tree for shelter. But the wind was fierce, and it tore her from the branch, and in spite of all her efforts bore her out over the ocean, farther and farther from the land, till at last, when its energy was spent, its fury gone, it left her on the ocean with no land in sight to guide her to her home, and as she flew she thought of her little ones at home, and of her mate. She thought she was flying to them, but every little effort was taking her farther away, though she knew it not. In her frightened cry I seemed to hear her say, 'O, when shall I rest my weary wing?' But in the murmuring of the ocean she heard no reply, so she could but fly on, till darkness came, when utterly exhausted, she fell upon the cruel waves and died. And He who made her will receive her when the course of life is past. Cannot the little robin find in that house of many mansions a place to rest her weary wing? Is heaven made for man alone? Are not these little creatures who never offend God, but worship Him with the purity and happiness of their little hearts entitled to the joys of the hereafter? Who can doubt it?"

